# Structural Analysis of a Repetitive Protein Sequence Motif in Strepsirrhine Primate Amelogenin

**DOI:** 10.1371/journal.pone.0018028

**Published:** 2011-03-18

**Authors:** Rodrigo S. Lacruz, Rajamani Lakshminarayanan, Keith M. Bromley, Joseph G. Hacia, Timothy G. Bromage, Malcolm L. Snead, Janet Moradian-Oldak, Michael L. Paine

**Affiliations:** 1 Center for Craniofacial Molecular Biology, Herman Ostrow School of Dentistry, University of Southern California, Los Angeles, California, United States of America; 2 Singapore Eye Research Institute, Singapore, Singapore; 3 Department of Biochemistry and Molecular Biology, University of Southern California, Los Angeles, California, United States of America; 4 Departments of Biomaterials and Biomimetics and Basic Science and Craniofacial Biology, New York University College of Dentistry, New York, New York, United States of America; Ecole Normale Supérieure de Lyon, France

## Abstract

Strepsirrhines are members of a primate suborder that has a distinctive set of features associated with the development of the dentition. Amelogenin (AMEL), the better known of the enamel matrix proteins, forms 90% of the secreted organic matrix during amelogenesis. Although AMEL has been sequenced in numerous mammalian lineages, the only reported strepsirrhine AMEL sequences are those of the ring-tailed lemur and galago, which contain a set of additional proline-rich tandem repeats absent in all other primates species analyzed to date, but present in some non-primate mammals. Here, we first determined that these repeats are present in AMEL from three additional lemur species and thus are likely to be widespread throughout this group. To evaluate the functional relevance of these repeats in strepsirrhines, we engineered a mutated murine amelogenin sequence containing a similar proline-rich sequence to that of *Lemur catta*. In the monomeric form, the MQP insertions had no influence on the secondary structure or refolding properties, whereas in the assembled form, the insertions increased the hydrodynamic radii. We speculate that increased AMEL nanosphere size may influence enamel formation in strepsirrhine primates.

## Introduction

Strepsirrhines are members of a suborder of primates that include lorises, galagos, and lemurs. They are characterized by anatomical features relevant to the dentition, such as the presence of a tooth comb and a distinct dental formula and morphology different to most other primate suborders, tarsiiformes and anthropoidea [1]. In addition, relative to similar-sized anthropoids, lemurs present a fast dental development with several species being born with the milk dentition partially or fully erupted [2], [3].

Tooth development is regulated by a set of conserved genes that determine the number, position, and types of teeth that develop in the oral cavity [4], [5]. Once the tooth follicle has advanced to the bell stage, epithelial cells from the inner enamel epithelium start to elongate and polarize. Soon after, these cells (ameloblasts) express enamel matrix proteins (EMPs) that regulate the development of enamel microstructure by forming an extracellular scaffold that guides mineral growth [6].

Amelogenin is the most abundant EMP during enamel development [7]. It is secreted into the extracellular space from the apical end of polarized ameloblast cells [8] where it undergoes self-assembly to form spherical structures referred to as nanospheres. These nanospheres are involved in controlling the enamel crystal habit [9], [10] by interacting along the *c*-axis of the growing crystallites to generate high-aspect crystallites [10] and bind them to one another [11]. Analyses of protein to protein interactions have shown that deletion of either the N- or C- terminus of the amelogenin peptide sequence affects the capacity to assemble into nanospheres [12], [13]. Recent *in vitro* studies have shown that single amino acid changes in the N-terminus of amelogenin can alter the secondary structure and refolding properties [14] and results in a profile consistent with human amelogenesis imperfecta. In addition, the loss of the self-assembly domains alters the grouping of these crystallites into the enamel rod, the basic building block of enamel [14], [15].

Studies in protein evolution have played a significant role in understanding tooth mineralization [16], [17]. Comparative analyses of the primary structure of amelogenin in various mammals indicate that the central region evolves at a higher rate than TRAP and acidic C-terminus and includes deletion or insertion of proline-rich repeats [18]. Recently, amelogenin has been classified as belonging to the family of Intrinsically Disordered Proteins (IDP) which lacks a well-defined 3D structure under native conditions and is typically flexible, extended, and has little secondary structure *in vitro* in the absence of partners [19]. It has been observed that tandem arrays of proline-rich repeats are prevalent in the primary structure of IDPs or Intrinsically Disordered Regions (IDR), the latter evolving to a considerable extent by expansion of such repeats [20]. As there is no specific function associated with IDPs, it has been suggested that their evolutionary rate is less constrained [21]. Computational analyses using disorder prediction algorithms have shown that the central region in mammalian amelogenins is significantly disordered [19]. Therefore, understanding the physicochemical properties of inserted sequences in the disordered proteins will clarify the contributions of these repeats to the evolution of mammalian dentition.

**Figure 1:**
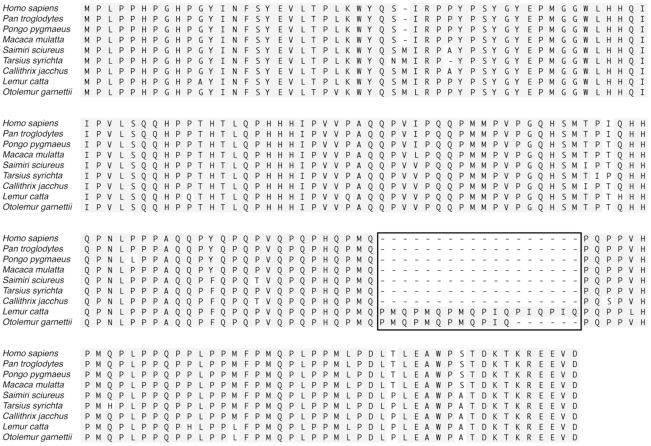
Clustal W alignment of primate amelogenin protein sequences derived from GenBank (see also **Table 1**). Strepsirhine primates (*Lemur catta* and *Otolemur garnettii*) contain multiple polyproline repeats not present in any other primate amelogenin sequences, as shown in the boxed area. A similar *L. catta* sequence of repeats was cloned into the mouse amelogenin cDNA backbone as detailed in the text.

Prior to this study, the available amelogenin sequences of two strepsirhine primates (*Lemur* and *Otolemur*) showed that they contained a number of tandem repeats in the central region, mainly proline (P), methionine (M) and glutamine (Q), about 12–18 amino acids long, which are not expressed in any other primate taxa [22] (**Figure 1**). Similar repeats are also found in other non-primate mammalian groups [18], [22]. It has been suggested that these repeats were likely part of the original amelogenin gene sequence, lost through evolutionary time in some lineages [18]. Delgado et al. (2005) [18] proposed that these tandem repeats are likely to play an important unspecificed role in tooth formation. These proline-rich tandem repeats may influence amelogenin's interactions with the mineral phase and affect dental development within strepsirrhines. Here, we report our initial investigation into the structural implications of amelogenin MQP repeat motif that is found in the strepsirrhine primates and several other non-primate mammalian lineages. Our results suggest that lemur enamel might have distinct properties from the enamel of other primates in which the repeats are absent.

## Materials and Methods

Amelogenin sequences available in GenBank include the following primate taxa (**Table 1**) (sequence numbers are included): human; chimpanzee (*Pan troglodytes*), orangutan (*Pongo pygmaeus*), rhesus macaque (*Macaca mulatta*), squirrel monkey (*Saimiri sciureus*), Philippine tarsier (*Tarsius syrichta*), common marmoset (*Callithrix jacchus*), ring-tailed lemur (*Lemur catta*), and small-eared galago (*Otolemur garnettii*). Protein and DNA sequences were analyzed using the ClustalW alignment algorithm and MacVector v. 2.1 software (MacVector). To further analyze whether these amino acid repeats are a widespread trait in lemurs, we included three additional species from two different families (Lemuridae and Daubentoniidae) and three different genera (*Daubentonia*, *Varecia* and *Eulemur*).

**Table 1:** Amelogenin sequences available for primates in GenBank.

Gene	Symbol	Species	NCBI Accession
**Amelogenin**	**AMELX**	*Homo sapiens*	AAC21581
		*Pan troglodytes*	ABQ50856
		*Pongo pygmaeous*	ABQ50857
		*Macaca mulatta*	ABQ50858
		*Saimiri sciureus*	BAC66103
		*Tarsius syrichta*	ABQ50860
		*Callithrix jacchus*	ABQ50859
		*Lemur catta*	BAC66105
		*Otolemur garnettii*	BAC66107

### DNA extraction, PCR and Sequencing

Whole-blood samples were obtained from the Duke Lemur Center for *Daubentonia madagascariensis* (Aye-aye); *Eulemur macaco flavifrons* (blue-eyed black lemur); and *Varecia varecia variegata* (black-and-white ruffed lemur). DNA extraction from blood was performed using PureLink Genomic DNA Mini Kit (Invitrogen), following manufacturer's specifications. Primers used to amplify AMEL exon 6, previously shown to contain the proline-rich repeat motif present in strepsirrhines, were designed based on human sequence information. Primer sequences are: Fw: 5′-AGCCTCATCACCACATCCCAGT-3′; and Rev: 5′-GGCAGGGGCTGCATGGGGA-3′. Touch-down PCR cycling was performed using TITANIUM™ Taq DNA Polymerase (Clontech) on a Biorad Mycycler as follows: 94°C (4 min) 94°C (30 sec) 67°C (30 sec) lowering 0.5 degrees 72°C (30 sec) ×10 cycles; 94°C (30 sec) 62°C (45 sec) 72°C (45 sec) ×25 cycles; 72°C (7 min). The expected product was 312 bp in length. For each species, the band of interest was subcloned into the pCR 2.1-TOPO vector (Invitrogen) and sequenced.

### Cloning strategy

The mouse amelogenin M180 peptide sequences and the corresponding lemur peptide sequence were aligned using ClustalW v. 2.1 (McVector v. 11.0.4) to identify the placement of the first and last residues of the repeats. The divergent sequence between mouse M180 and that of lemur was identified as four sets of the residues MQP, followed by a single isoleucine. It should be noted that the alignments obtained in our study by direct comparison of the mouse M180 and *L. catta* sequences differ from previous reports in which using a different alignment algorithm and software, the amelogenin sequences of 25 mammals were aligned [18], [22] (See Supporting Information).

Two recombinant proteins containing the poly-histidine N-terminal tag were prepared using the plasmid pQE30 (Qiagen Inc.) as the vector backbone. The first recombinant protein was to generate a His-tagged mouse M180 protein, a product that has been reported previously and identified as rp(H)M180 [23]. The second recombinant protein was essentially the same as for rp(H)M180 with the inclusion of the peptide (MQP)_4_I. This 203 amino acid long mouse-MQP chimeric amelogenin protein containing the ring-tailed lemur repeat sequence will be referred to as a “chimeric” protein.

Briefly, a PCR-based strategy was used to amplify 2 DNA amelogenin fragments (N-terminal and C-terminal) that could be ligated together. The rp(H)M180 vector served as template DNA for the PCR, and primers were synthesized such that the repeat region was included at the 3′-ends of the two primers that were used to generate the 3′-end of the first fragment, and the 5′-end of the second fragment. The entire cDNA region of the newly created rp(H)M180L plasmid was sequenced to ensure no PCR-introduced errors, or cloning artifacts, were introduced.

### Protein purification

Recombinant proteins rp(H)M180 and the chimeric protein were prepared using the expression vector pQE30 (Qiagen Inc.), expressed in *E. coli*, isolated, and purified using QIAexpress Ni-NTA Protein Purification System following previously described protocols [23].

### Disorder Prediction

The normalized values of the Kyte & Doolittle hydrophobicity scale for individual residues were obtained using the ExPASy Proteomics Server, http://www.expasy.org/tools/protscale.html. The mean hydrophobicity is the sum of the hydrophobicity of all amino acid residues divided by the total number of residues and mean net charge is the absolute net charge at pH 7.0 divided by total number of residues. The degree of disorder was calculated based on the PONDR (prediction of natural disordered regions) with standard parameter settings [24].

### Circular dichroism (CD)

CD spectropolarimetry is a technique commonly used to investigate the secondary structure of proteins by differential absortion of right-left circularly polarized light. CD experiments were performed on a Jasco J-810 (and J-815) spectropolarimeter equipped with Peltier set up. The proteins were prepared to a concentration of 0.4 mg/mL and dissolved in 25 mM buffer at pH 5.8±0.1 (sodium acetate) or pH 8±0.1 (Tris-Cl) at 25**°**C. At pH 5.8 amelogenins do not assemble into nanospheres, but they do so at pH 8. A Suprasil quartz cell with a path length of 1 or 0.1 mm was used. For the wavelength scan, spectra were monitored between 190-240 nm with a resolution of 0.1 nm and a band width of 2 nm. For the variable temperature CD spectra was performed from 10 – 30°C at 5°C intervals, after equilibrating the proteins for 10 minutes at each temperature. The final spectra reported were an average of 16 scans. All the spectra were background subtracted and smoothened by Savitzky-Golay method using a window size of 5 nm. For the single wavelength measurements, the proteins were heated/cooled (10-70°C) at 5°C/min and the change CD intensity at 224 nm was monitored. For the refolding experiments, proteins were heated to 70°C, kept at that temperature for 5 min before being cooled to 25°C and the changes in CD intensity at 200 nm was monitored as a function of time.

### Dynamic light scattering (DLS)

DLS is commonly used to assess the size distribution of particles in solution. Measurements of the hydrodynamic radii of the chimeric and rp(H)M180 amelogenin nanospheres (0.4 mg/mL, pH 8, 25 mM Tris buffer) were performed using a Wyatt DynaPro Nanostar dynamic light scattering instrument (Wyatt Technology). Experiments were performed at 22°C. The acquisition time was 10 seconds and 10 acquisitions were collected to complete one measurement (100 seconds total measurement time). Thirty measurements were recorded for each protein sample. The data were analyzed using Dynamics 7.0 software. The dynamic light scattering data were produced by the program performing a regularization fit using the Dynals algorithm on the resultant autocorrelation functions. A Rayleigh sphere model was used for the analysis meaning that the hydrodynamic radii calculated were sphere-equivalent radii.

### Fluorescence anisotropy

Anisotropy experiments of the chimeric lemur and mouse amelogenin nanospheres (0.4 mg/mL, pH 8, 25 mM Tris-Cl buffer) were carried out at 22°C using a Photon Technology International (PTI) QuantaMaster20. The excitation wavelength was 295 nm and the emission wavelength was detected at 336 nm. Forty “ten second” acquisitions were taken and averaged to provide the final value and standard deviation. The results were analyzed using PTI Felix32 software.

## Results

### Cloning of lemur exon 6

**Figure 2:**
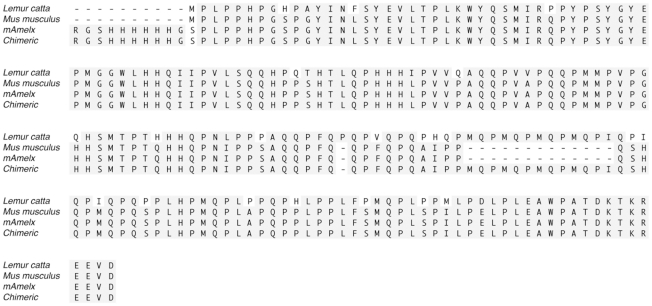
ClustalW alignments for the *Lemur catta* and *Mus musculus* X-derived amelogenin protein, and the recombinant proteins used to study the role of the MQP repeats sequences in exon 6. Previously, the mouse cDNA backbone sequence was used to create a recombinant protein [rp(H)M180] identified here as mAmelx. Using rp(H)M180 as template DNA, a PCR-based strategy was used to create a recombinant chimeric protein that had four MQP repeats added at the region shown (bottom line). We refer to this as the mouse-lemur chimeric protein. It should be noted that the alignments presented here are based on using ClustalW version 2.1 in MacVector version11.0.4 software aligning the *Lemur catta* sequence (EU168853) and that of *Mus musculus* (NP_033796). The resulting aligned sequence of these two species differs from those reported previously which used a different alignment algorithm and software [18], [22]. However, this difference does not affect the analyses performed in this study of the purified proteins.

**Figure 3:**
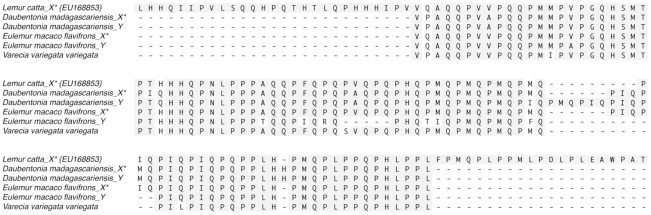
ClustalW alignment of amino acid sequences of the newly cloned lemur exon 6 amelogenin. The derived amino acid sequence based on DNA sequence data from either female (XX) or male (XY) whole blood samples. X-derived or Y-derived AMEL sequences are assigned for *Daubentonia* and *Eulemur*. For *Varecia*, the sequence is derived from a male individual according to the records from the Duke Lemur Center. However, we had only limited DNA sample available and although we were able to amplify one product of the *V. varecia* sample, we could not confirm whether this was X- or Y-derived. (*) indicates multiple PCR and sequencing, which confirmed that there were no PCR introduced errors.

**Figure 2** shows the ClustalW alignments for the *Lemur catta* and *Mus musculus* X-derived AMEL, and the recombinant proteins used to study the role of the MQP repeats sequences present in exon 6**.** **Figure 3** shows the translated protein of the partial sequence of amelogenin exon 6 from three new lemur species (*Varecia v. variegata*, *Eulemur macaco flavifrons* and *Daubentonia madagascariensis*) showing that all lemur species sampled to date contain within the exon 6 of amelogenin a number of MQP repeats. However, some differences were noted between the AMELX and AMELY derived lemur amelogenin sequences (**Figure 3**).

### Recombinant mouse AMEL protein containing lemur MQP repeats

All primate amelogenin proteins are enriched in amino acid residues that promote disorder and depleted in amino acid residues that confer structure to a protein [19]. The structural disorder/order in the recombinant mouse and chimeric (mouse-MQP) amelogenin was examined using the mean net charge versus mean hydrophobicity plot that allows the binary classification of proteins [25]. Analysis of the amelogenin from *H. sapiens* and *L. catta* show that both occupy non-overlapping intrinsically disordered region of the plot (data not shown). Wild type mouse amelogenin and mouse-lemur chimera also occupy the intrinsically disordered region indicating that the properties of the two amelogenins are similar to those of intrinsically disordered proteins.

### Variable Temperature CD Spectra

**Figure 4:**
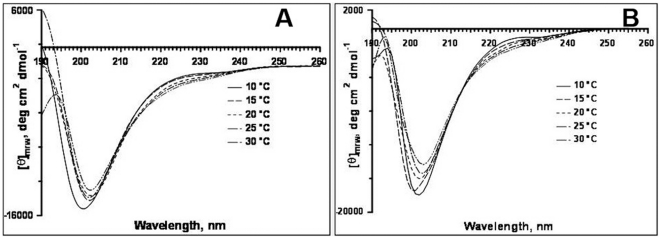
Secondary structure of amelogenins. VT-CD spectra of mouse (A) and chimeric (B) amelogenins in 25 mM sodium acetate buffer (pH = 5.8) and mouse amelogenin. The CD spectra of the wild type mouse and the chimeric proteins exhibited all the hallmarks of an intrinsically disordered proteins i.e., a strong minimum around 202 nm and a weak shoulder in the π-π* region, an increase in the CD intensity in the π-π* region with temperature, and a well-defined iso-elliptic point around 211–213 nm.

To test the prediction experimentally and how the -MQP- repeats influence the degree of disorder, we characterized the secondary structure of the wild type and mouse-lemur amelogenin by far UV-CD spectroscopy. **Figure 4** shows the VT-CD spectra of the wild type mouse and mouse-MQP amelogenin monomers at pH 5.8. The CD spectra of the wild type mouse and the chimeric proteins exhibited all the hallmarks of an intrinsically disordered protein i.e., a strong minimum around 202 nm and a weak shoulder in the π-π* region, an increase in the CD intensity in the π-π* region with temperature, and a well-defined iso-elliptic point around 211-213 nm. At 25°C, the difference spectrum (obtained by subtracting the CD spectrum of wild type mouse amelogenin from the mouse-MQP protein) indicates the incorporation of non-conserved -MQP- motifs in the chimeric mouse-MQP amelogenin increase the degree of disorder.

**Figure 5:**
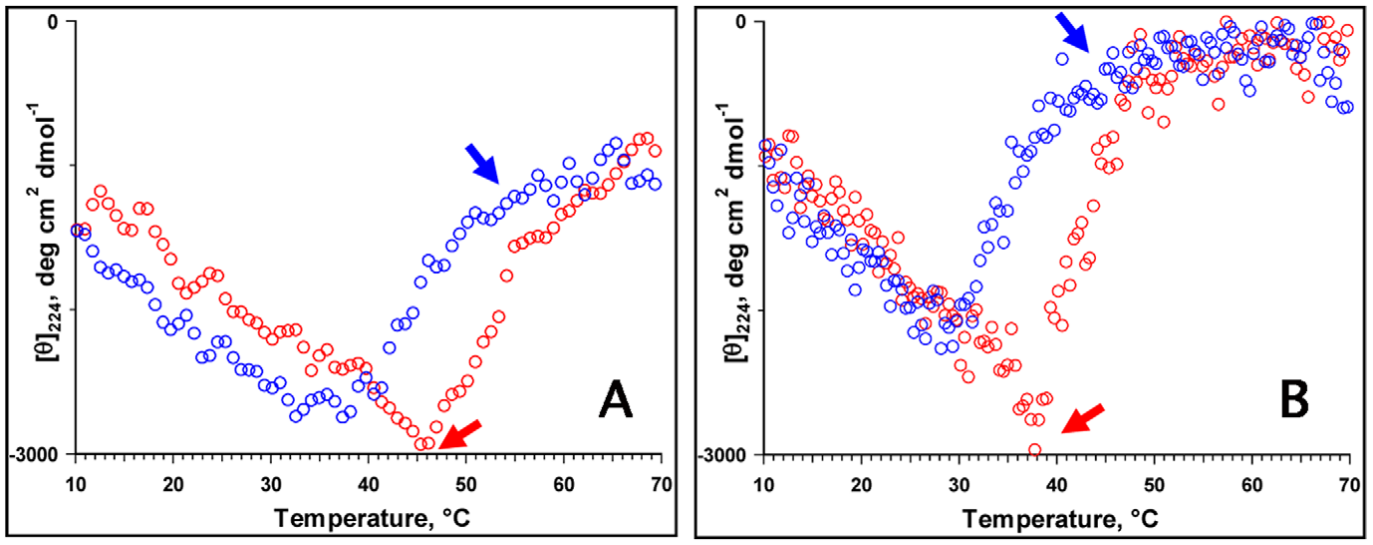
Thermal unfolding-refolding behavior of mouse (A) and chimeric (B) amelogenins. The figure represents change in ellipticity at 224 nm as a function of temperature. The heating and cooling cycles are represented by red and blue open circles, respectively. Note that the –MQP- inserted chimeric amelogenin undergoes similar biphasic transitions as the mouse amelogenin. The onsets of unfolding and refolding are indicated by red and blue arrows, respectively.

To assess the role of -MQP- repeats, we have monitored the effect of temperature on the CD ellipticity at 224 nm for the recombinant mouse and the chimeric protein. **Figures 5A and 5B** compare the change in [θ]_224_ as a function of temperature for mouse amelogenin and the chimera. At low temperatures, the CD intensity increased with temperature indicating a non-cooperative unfolded-to-folded transition. Above 45°C, denaturation began with a sharp reduction in CD intensity (indicated by red arrow in **Figure 5B**), as has been observed for folded proteins. On cooling (blue open circles), the CD intensity retraced back slowly and the CD intensity began to increase at 45°C (indicated by red arrow in 5B). Complete refolding occurred at around 41°C (indicated by blue arrow in **Figure 5A**) and the CD intensity followed the same path as the heating cycle upon further cooling. Thus, rM180 exhibited a weak hysteresis upon denaturation (heating) and refolding (cooling).

The chimeric protein also exhibited a chevron shaped curve on heating. However, the temperature at which changes in CD intensity observed was different from mouse amelogenin. On heating, the CD intensity at 224 nm increased gradually and reached a maximum around 40°C, above which the intensity decreased sharply decreased and reached minimum above 45°C. On cooling, no significant change was observed until 38°C. Below this temperature, the CD intensity began to increase and reached maximum value around 30°C. Further cooling led to a gradual decrease in the CD intensity.

**Figure 6:**
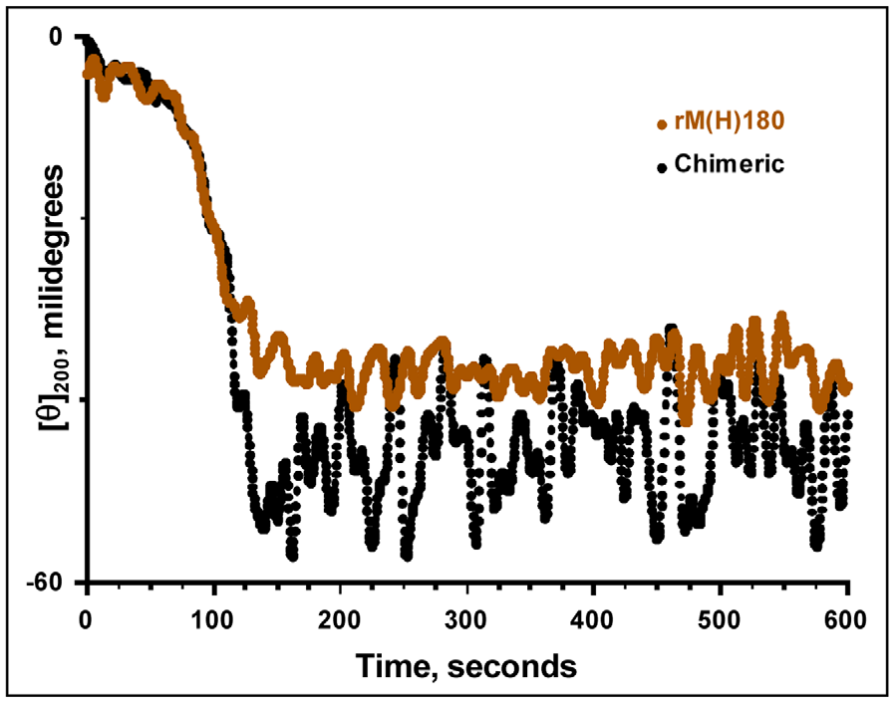
Refolding kinetics of mouse and the chimeric amelogenins. The proteins were denatured by heating the 0.4 mg/mL solution to 70°C and cooled to 25°C rapidly. The change in CD intensity at 200 nm was monitored as a function of time. Note that both the proteins reached the CD intensity at 25°C very rapidly. The t_1/2_ value (which is defined as the time required to reach 50% CD intensity at 200 nm) for the chimeric amelogenin is 104 seconds whereas for the mouse amelogenin is 98.5 seconds.

To obtain a further insight into the refolding process, we have also studied the kinetic changes in CD intensity at 200 nm. In these experiments, the protein was heated to 70°C and quenched quickly to 25°C. The change in CD intensity at 200 nm is monitored as a function of time. **Figure 6** compares the refolding behavior of mouse amelogenin and the chimera. Both the wild type and the chimera have similar t_1/2_ values i.e. the time required to reach the 50% of the initial conformation and kinetic rate constants.

### Assembled form

**Figure 7:**
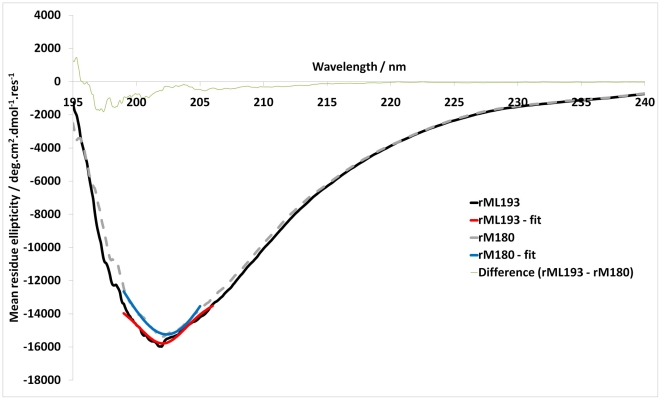
CD spectra of mouse and chimeric amelogenins at pH 8. No difference spectrum was observed between the chimeric protein (rM180 containing *L. catta* repeats) and the wild type rp(H)M180.

**Figure 8:**
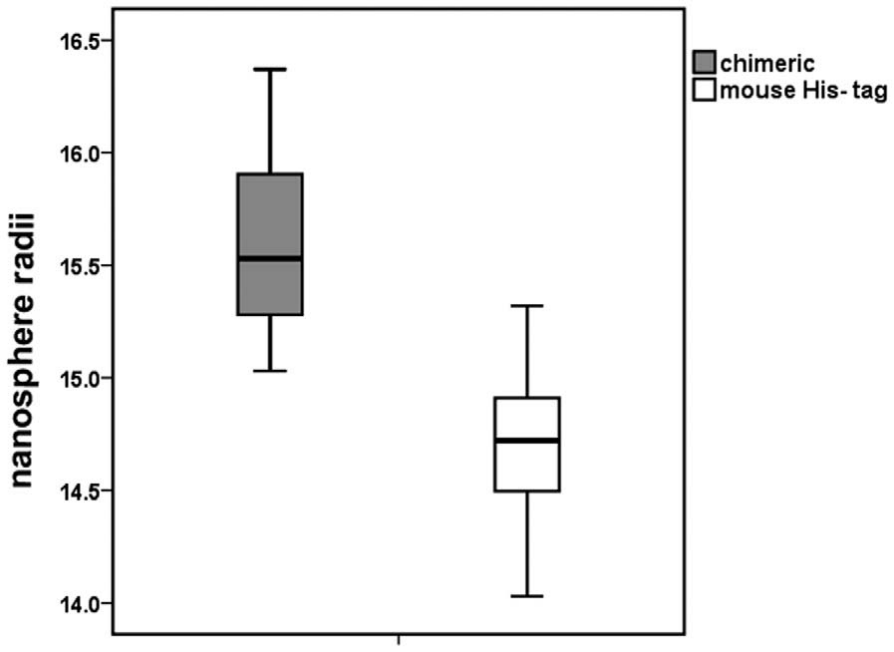
Box plot of measurements taken using DLS of nanosphere radii from the mouse rp(H)M180 and the chimeric protein. The means of wild type and chimeric radii differ significantly (p<0.001).

To infer the similarity/differences we have also investigated the self-assembly properties of the chimeric and mouse amelogenin. When the CD spectra of wild type mouse and mouse-MQP chimeric amelogenin were recorded at pH 8 wherein the amelogenin exists in an assembled form, no significant difference was observed (**Figure 7**). However, results from the DLS analysis of the radii of nanospheres from wild type mouse (rp(H)M180) compared with the mouse- MQP protein indicate that the average radii of nanospheres formed from the chimeric protein containing the repeats increases by ∼6% compared to wild type rp(H)M180 (see **Table 2**). **Figure 8** shows box plots of the distribution of values for nanosphere radii in rp(H)M180 mouse amelogenin and the mouse-MQP protein. Statistical comparison of the means using two independent samples t-test shows highly significant differences between the means (p<0.001).

**Table 2:** Average of nanosphere radii in rp(H)M180 and chimeric proteins.

Protein type	Mean	SD
rp(H)M180 (wild type protein)	14.7	0.33
chimeric	15.6	0.36
**p<0.001**		

The difference in radii is significant using student's t-test (samples were normally distributed).

Fluorescence anisotropy measurements were also performed in order to confirm the finding that nanospheres had increased in size. In this instance, the tryptophan amino acids within the amelogenin sequence were used as the fluorophore (λ_ex_ 295 nm, λ_em_ 336 nm). The mouse-MQP amelogenin nanospheres had a larger anisotropy (0.111±0.0009) than wild type mouse amelogenin nanospheres (0.108±0.0009), supporting the DLS evidence that the chimeric amelogenin nanospheres are larger.

## Discussion

Primate tooth shape and size, and enamel microstructure and thickness, are key characters commonly used to reconstruct the life history and evolutionary place of extant and extinct primates [26]. To understand the functionality of teeth, it is essential to investigate the hierarchical developmental events that generate the final tooth shape that will be used by an individual to survive in its environment and that will characterize its own life history. The assembly of EMPs is critical to this process. By interacting with growing crystals, EMPs can generate one of the most complex biological structures in nature. Although proline-rich repeats are present in the amelogenin sequence of a number of diverse non-primate mammalian taxa [18], we focused our study on primates since they include the largest number of species for which the amelogenin sequence is known. Amongst primates, only strepsirrhines possess the repeats of interest. Furthermore, they have unique dental characteristics relative to all other primates [3] and are considered by some to represent a basal primate lineage [27], reflecting their relevance in understanding the evolution of their unique dental development (see below).

Lemurs are restricted to the island of Madagascar, where at present, five extant families (Cheirogaleidae; Lemuridae; Lepilemuridae; Indriidae and Daubentoniidae) which include 14 genera and as many as 32 species, are recognized [28]. The current understanding on the lemur habitation of Madagascar, based on divergence age analyses, has estimated that lemurs did not arrive in Madagascar until ∼50 Mya [29], postdating the geographical separation of this island from the African continent [30]. They also present unique characteristics and evolutionary adaptations in their dental development. Madagascar lemurs, extant and extinct species, differ from the haplorrhines in their rapid dental development [2], [3]. Among the extant Malagasy lemurs, members of the family *Indridae* are born with the milk dentition erupted, whereas other lemur species achieve eruption of the permanent teeth by year one [2], [3]. This is in stark contrast with haplorrhines, a group that overall displays a more delayed dental development [3].

The full-length human amelogenin (*AMEL*) gene transcript is ∼800 bp long, and encodes for 191 amino acids. Amelogenin may contain up to 9 exons in rodents [31], but most commonly only 7 exons are recognized [32]. In primates, but not in mice, there are two *AMEL* loci with one copy of the gene on each of the sex chromosomes (AMELX and AMELY) [33].

Given the critical importance of EMPs in the correct development of enamel, we investigated the amelogenin protein structure of strepsirrhines using the mouse amelogenin cDNA backbone.

Here, we cloned a partial region of exon 6 of three lemur species to investigate the possibility that all lemur amelogenin proteins contain additional amino acid repeats rich in proline. Our results show that all lemur species analyzed to date contain tandem repeats in the central region of amelogenin. We also generated and characterized the structure of a mouse- chimeric amelogenin containing the MQP repeats. We used a well characterized mouse amelogenin protein (rp(H)M180; [23] and a variant of the exact same protein which differed only in that the latter contained four MQP repeats similar to those found in the lemur amelogenin sequence.

In the monomeric form (i.e. at pH 5.8), the MQP insertions leads to more disorder in the secondary structure of the chimeric protein as indicated by a large increase in CD intensity at 202 nm. VT-CD studies (**Figure 4**) suggested that the chimeric protein exists in an unordered conformation in equilibrium with PPII structure at lower temperature and formed ordered conformations at higher temperatures. These are characteristic features of natively unfolded or intrinsically disordered proteins. Similar to the mouse amelogenin, the thermal behavior of chimera is biphasic i.e. it involves two transitions. From 10 to 40°C, the transition is non-cooperative linear thermal transition as has been observed for IDPs. From 40 to 70°C, the transition is co-operative as has been observed for a folded protein. The non-cooperative transition is accompanied by changes from an intrinsically disordered structure to a partially folded structure which is converted into an aggregated structure during co-operative transition upon further heating. The weak hysteresis observed in the thermal denaturation of mouse amelogenin still persisted in the mouse-MQP amelogenin. This further confirms our experimental data that the insertion had no influence on the secondary structure. Refolding kinetics also demonstrated that both mouse and the chimera reached the initial conformation in an almost identical manner indicating no apparent changes in the refolding kinetics as a result of the insertion.

**Figure 9:**
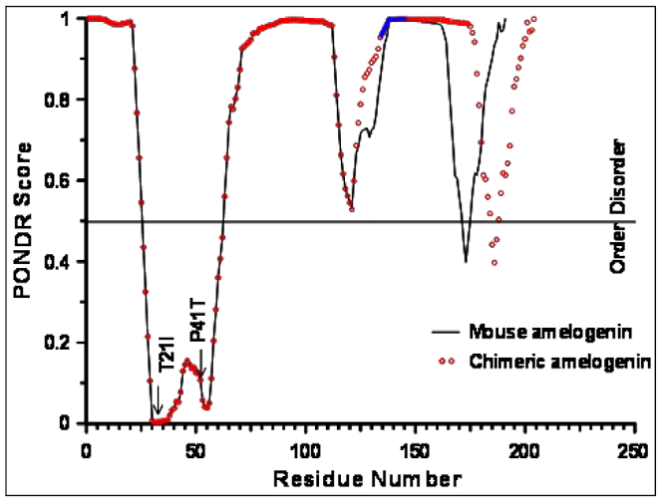
PONDR prediction of intrinsic disorder regions in the recombinant mouse and the chimeric amelogenins. The predictions of disorder (scores greater than 0.5) and order (scores less than 0.5) is plotted against the residue number. Note that the insertion of MQP repeats in the mouse increased the disorder propensity of the mouse amelogenin. The two amelogenesis imperfecta variants (T21I and P41T), which altered the thermal properties of wild type amelogenin significantly, are also shown to indicate that any alterations in the “ordered” regions significantly affect the thermal refolding properties. The solid blue line indicates MQP insertion.

To obtain a better insight into the structure-function relationship, we analyzed the results based on PONDR (Predictors of Natural Disordered Regions) methods for predicting the disorder regions (DRs) in proteins [24]. As shown in **Figure 9**, the insertion enhances the disorder propensity (shown as solid blue lines) in the mouse amelogenin. Recently, we have shown that single amino acid substitutions in the tyrosine-rich amelogenin polypeptide (TRAP) domain facilitates misfolding or oligomerization of recombinant rp(H)M180 [34]. Thus, it is possible that alterations in DRs may have little influence on the properties of the amelogenin monomers.

We found that the nanosphere radii significantly increased in the mouse-MQP protein relative to the rp(H)M180 mouse amelogenin (p<0.001) (**Figure 8**). The increase in nanosphere size with increase in repeats observed in our study (demonstrated by both DLS and fluorescence anisotropy) is at odds with previous findings [35]. Using model peptides, it was shown that an increase in proline-rich repeats decreased the size of nanospheres assembly and increased the enamel crystal length [35]. These authors [35] used a mixture of EMPs extracted from unerupted teeth of amphibians and mouse, which do not contain the repeats, and unerupted teeth of goat and cow, which do contain these repeats in variable numbers. The cow sequence had the highest number of repeats of the species used in their study. Supramolecular assembly of these proteins *in vitro* produced amelogenin nanospheres which were measured by Atomic Force Microscopy (AFM) and DLS. The average diameters of the nanospheres decreased with increased numbers of repeats, so that cow, with the highest number of repeats, displayed the smallest nanosphere diameter. Whereas Jin et al. (2009) [35] extracted native proteins from the enamel of a variety of species with unique sequences which are in part, but no wholly, characterized by these amino acid repeats, and analyzed their supramolecular assembly; our study compared two identical proteins that differed only in that the mouse-lemur chimeric protein included four MQP repeats. Therefore, no differences in the N- or C- terminus of the amelogenins, nor differences in the presence of any other enamel proteins that may have been included in the processing of native proteins from Jin et al. (2009) [35], can account for the differences in nanosphere size that we have detected in our study. In summary, differences in DLS results between studies likely derive from using a crude mixture of amelogenin and amelogenin proteolytic products [35], and the use of purified recombinant protein to measure nanospheres (this study).

To further characterize the properties of our mouse-chimeric protein, we investigated the possibility that the folding of this protein was affected by using CD spectra analysis of mouse rp(H)M180 and mouse-MQP amelogenin measured at pH 8. Our results show that the increased flexibility observed when the amelogenins were in the monomeric form was lost when they were assembled into nanospheres (pH 8) (**Figure 7**).

Although we note structural changes (i.e. increase nanosphere radii), the functional consequences of MQP repeats in strepsirrhine AMEL proteins remain to be elucidated. Recent research carried out in our laboratory has shown that two transmembrane proteins involved in matrix endocytosis (CD63 and LAMP -lysosome-associated membrane protein-), interact with amelogenin at specific proline rich domains [36]. The exogenous addition of amelogenin in cell cultures shows that this protein is rapidly moved into CD63/LAMP1 positive vesicles [37]. Hence, it may be suggested that proline rich regions of amelogenin, such as those linked to the lemur amelogenin, may play a role in the ability of ameloblasts to more rapidly endocytose the cleaved amelogenin fragments after proteolytic processing of this protein, facilitating mineralization. It has also been suggested that proline-rich repeats are a common feature in biomineralizing organisms and their putative functions include protein-protein interactions and a role as a mineral-binding domain [38]. Also, it was demonstrated that these repeats affect crystal growth by increasing crystal size [35].

Collectively, the increased nanosphere radii in the mouse-MQP amelogenin, putative associations with more efficient endocytotic processing, enhanced mineral binding of proline-rich proteins, and increased crystal length, suggest that strepsirrhine AMEL may have distinct properties absent in primate orthologs without the repeats. Although other important factors (e.g. genetic regulation, signaling roles of amelogenin [39], [40]) have significant functions in enamel development, this study focused on the structural analysis of amelogenin MQP repeats.

### Conclusions

Additional studies of the strepsirrhine AMEL protein will be needed in order to determine to what extent (if any) the polyproline repeats influence dental phenotypes in this primate group. Given that genetic engineering methods are not available in strepsirrhine primates, we propose that the investigation of chimeric AMEL proteins expressed in mice provides a novel method of addressing these questions. Here, we demonstrate that the chimeric mouse protein has distinct structural characteristics relative to that of wild type mouse protein. The dental phenotypes of genetically engineered mouse models expressing chimeric AMEL proteins could provide novel insights on the evolution of dentition in human and non-human primates.

## References

[1] Fleagle JG (1998).

[2] Schwartz GT, Samonds KE, Godfrey LR, Jungers WL, Simons EL (2002). Dental microstructure and life history in subfossil Malagasy lemurs.. Proceedings of the National Academy of Sciences of the United States of America.

[3] Godfrey LR, Samonds KE, Wright PC, King SJ (2005). Schultz's Unruly Rule: Dental Developmental Sequences and Schedules in Small-Bodied, Folivorous Lemurs.. Folia Primatologica.

[4] Tucker A, Sharpe P (2004). The cutting-edge of mammalian development; how the embryo makes teeth.. Nat Rev Genet.

[5] Thesleff I (2006). The genetic basis of tooth development and dental defects.. American Journal of Medical Genetics Part A.

[6] Paine ML, White SN, Luo W, Fong H, Sarikaya M (2001). Regulated gene expression dictates enamel structure and tooth function.. Matrix Biology.

[7] Simmer JP, Fincham AG (1995). Molecular mechanisms of dental enamel formation.. Crit Rev Oral Biol Med.

[8] Boyde A, Berkovitz B (1989). Enamel.. Teeth: Handbook of Microscopi Analysis.

[9] Moradian-Oldak J (2001). Amelogenins: assembly, processing and control of crystal morphology.. Matrix Biology.

[10] Fincham AG, Moradian-Oldak J, Simmer JP, Sarte P, Lau EC (1994). Self-Assembly of a Recombinant Amelogenin Protein Generates Supramolecular Structures.. Journal of Structural Biology.

[11] Moradian-Oldak J, Tan J, Fincham AG (1998). Interaction of amelogenin with hydroxyapatite crystals: an adherence effect through amelogenin molecular self-association.. Biopolymers.

[12] Paine ML, Krebsbach PH, Chen LS, Paine CT, Yamada Y (1998). Protein-to-protein interactions: criteria defining the assembly of the enamel organic matrix.. J Dent Res.

[13] Moradian-Oldak J, Paine ML, Lei YP, Fincham AG, Snead ML (2000). Self-assembly properties of recombinant engineered amelogenin proteins analyzed by dynamic light scattering and atomic force microscopy.. J Struct Biol.

[14] Zhu D, Paine ML, Luo W, Bringas P, Snead ML (2006). Altering biomineralization by protein design.. J Biol Chem.

[15] Zhu DH, Paine ML, Luo W, Laborde JN, Bringas P, Snead ML, Sodek WLJ (2004). Engineered amelogenin domains expressed from a genetic knock-in locus: Analysis of herterozygous female incisors..

[16] Kawasaki K, Weiss KM (2003). Mineralized tissue and vertebrate evolution: The secretory calcium-binding phosphoprotein gene cluster.. Proceedings of the National Academy of Sciences of the United States of America.

[17] Kawasaki K, Suzuki T, Weiss KM (2005). Phenogenetic drift in evolution: The changing genetic basis of vertebrate teeth.. Proceedings of the National Academy of Sciences of the United States of America.

[18] Delgado S, Girondot M, Sire J-Y (2005). Molecular Evolution of Amelogenin in Mammals.. Journal of Molecular Evolution.

[19] Moradian-Oldak J, Lakshminarayanan R, Goldberg M (2010).

[20] Tompa P (2003). Intrinsically unstructured proteins evolve by repeat expansion.. BioEssays.

[21] Brown CJ, Takayama S, Campen AM, Vise P, Marshall TW (2002). Evolutionary Rate Heterogeneity in Proteins with Long Disordered Regions.. Journal of Molecular Evolution.

[22] Sire J-Y, Delgado S, Fromentin D, Girondot M (2005). Amelogenin: lessons from evolution.. Archives of Oral Biology.

[23] Moradian-Oldak J, Paine ML, Lei YP, Fincham AG, Snead ML (2000). Self-assembly properties of recombinant engineered amelogenin proteins analyzed by dynamic light scattering and atomic force microscopy.. J Struct Biol.

[24] Obradovic Z, Peng K, Vucetic S, Radivojac P, Brown C (2003). Predicting intrinsic disorder from amino acid sequence.. Proteins.

[25] Uversky VN, Gillespie JR, Fink AL (2000). Why are “natively unfolded” proteins unstructured under physiologic conditions?. Proteins.

[26] Lacruz RS (2007). Enamel microstructure of the hominid KB 5223 from Kromdraai, South Africa.. Am J Phys Anthropol.

[27] Purvis A (1995). A Composite Estimate of Primate Phylogeny.. Philosophical Transactions of the Royal Society of London Series B: Biological Sciences.

[28] Pastorini J, Thalmann U, Martin RD (2003). A molecular approach to comparative phylogeography of extant Malagasy lemurs.. Proceedings of the National Academy of Sciences of the United States of America.

[29] Yoder AD, Yang Z (2004). Divergence dates for Malagasy lemurs estimated from multiple gene loci: geological and evolutionary context.. Molecular Ecology.

[30] Kappeler PM (2000). Lemur Origins: Rafting by groups of hibernators?. Folia Primatologica.

[31] Papagerakis P, Ibarra JM, Inozentseva N, DenBesten P, MacDougall M (2005). Mouse Amelogenin Exons 8 and 9: Sequence Analysis and Protein Distribution.. Journal of Dental Research.

[32] Salido E, Yen PH, Koprivnikar K, Yu LC, Shapiro J (1992). The Human Enamel protein gene Amelogenin is Expressed from Both the X and the Y Chromosomes.. American Journal of Human Genetics.

[33] Lau EC, Mohandas TK, Shapiro LJ, Slavkin HC, Snead ML (1989). Human and mouse amelogenin gene loci are on the sex chromosomes.. Genomics.

[34] Lakshminarayanan R, Bromley KM, Lei Y-P, Snead ML, Moradian-Oldak J (2010). Perturbed Amelogenin Secondary Structure Leads to Uncontrolled Aggregation in Amelogenesis Imperfecta Mutant Proteins.. Journal of Biological Chemistry.

[35] Jin T, Ito Y, Luan X, Dangaria S, Walker C (2009). Elongated Polyproline Motifs Facilitate Enamel Evolution through Matrix Subunit Compaction.. PLoS Biol.

[36] Zou Y, Wang H, Shapiro JL, Okamoto CT, Brookes SJ (2007). Determination of protein regions responsible for interactions of amelogenin with CD63 and LAMP1.. Biochem J.

[37] Shapiro J, Wen X, Okamoto C, Wang H, Lyngstadaas S (2007). Cellular uptake of amelogenin, and its localization to CD63, and Lamp1-positive vesicles.. Cellular and Molecular Life Sciences.

[38] Zhang B, Xu G, Evans JS (2000). Model peptide studies of sequence repeats derived from the intracrystalline biomineralization protein, SM50. II. Pro,Asn-Rich tandem repeats.. Biopolymers.

[39] Veis A, Tompkins K, Alvares K, Wei K, Wang L (2000). Specific Amelogenin Gene Splice Products Have Signaling Effects on Cells in Culture and in Implants in Vivo.. Journal of Biological Chemistry.

[40] Warotayanont R, Frenkel B, Snead ML, Zhou Y (2009). Leucine-rich amelogenin peptide induces osteogenesis by activation of the Wnt pathway.. Biochemical and Biophysical Research Communications.

